# Rhizoma Pinelliae trypsin inhibitor separation, purification and inhibitory activity on the proliferation of BGC-823 gastric adenocarcinoma cells

**DOI:** 10.3892/etm.2014.1701

**Published:** 2014-05-08

**Authors:** GUOHONG ZU, HOUWEI WANG, JIE WANG, YAN DOU, WEICHONG ZHAO, YUPING SUN

**Affiliations:** 1 Department of Radiation Oncology, Jinan Central Hospital Affiliated to Shandong University, Jinan, Shandong 250013, P.R. China; 2 Department of Chinese Pharmacy, Shandong University of Traditional Chinese Medicine, Jinan, Shandong 250013, P.R. China; 3 Department of Surgery, Jinan Central Hospital Affiliated to Shandong University, Jinan, Shandong 250013, P.R. China

**Keywords:** *Pinellia*, trypsin inhibitor, human poorly differentiated gastric adenocarcinoma

## Abstract

The aim of this study was to isolate and purify Rhizoma Pinelliae trypsin inhibitor (RPTI), determine its N-terminal amino acid sequence and evaluate its inhibitory effect on the proliferation of poorly differentiated BGC-823 human gastric adenocarcinoma cells. RPTI was separated and purified from a 40% (NH_4_)_2_SO_4_ precipitate of crude protein extract of *Pinellia ternata* tuber using affinity chromatography with trypsin as the ligand. The N-terminal amino acid sequence of RPTI was determined using the Edman degradation method. The inhibitory effect of RPTI on BGC-823 cell proliferation was detected *in vitro* using the MTT method and *in vivo* in tumour-bearing mice. The purified RPTI showed a single band under SDS-PAGE, its molecular weight was 14 kDa and its N-terminal amino acid sequence was DPVVDG. RPTI inhibited trypsin activity, with an inhibition ratio of 1:6.78 (mass). RPTI significantly inhibited the proliferation of BGC-823 cells *in vitro*. The IC_50_ of RPTI was 16.96 μg/ml within 48 h after treatment and 9.61 μg/ml within 72 h after treatment. Subcutaneous injection of RPTI around the tumour significantly inhibited BGC-823 tumour growth in mice. The tumour inhibitory effect was concentration- and dose-dependent. RPTI did not significantly influence the spleen coefficient of the mice. In conclusion, RPTI is a serine proteinase inhibitor with antitumour activity.

## Introduction

The traditional Chinese herbal medicine Rhizoma Pinelliae (RP) is the tuber of *Pinellia ternata* (Thunb.) Breit., and is listed in the Dictionary of Traditional Chinese Medicine (1) as effective in removing dampness to reduce phlegm, reducing adverse qi for controlling nausea and vomiting, and relieving distension to eliminate stagnation, among other effects (2–4). RP is used alone or in combination with other Chinese medicines to treat various neoplastic diseases. This plant is also used as folk medicine for treating cancer in several regions of China (5–8). There have been a number of studies on the antitumour activity of *Pinellia*, such as the inhibitory effects of ethanolic *Pinellia* extract on liver cancer cells, of total organic acids of *Pinellia* on gastric cancer cells and of *Pinellia* protein on ovarian cancer cells (8–12). However, to the best of our knowledge, the antitumour activity of low molecular weight components of *Pinellia* has not been reported. Trypsin inhibitors (TIs), low molecular weight proteins that inhibit various serine proteinases, are widely present in animals, plants and microorganisms. TIs inhibit the catalytic activity of enzymes or prevent zymogen activation through combination with the active and allosteric site of the proteinase. TIs are crucial in regulating physiological and pathological processes and are an important component of the immune system. TIs have extensive application prospects in the research and development of antitumour drugs (13–18). Studies have demonstrated that TI receptors are present in numerous types of tumour cell, and TIs exert their antitumour function by binding with the receptors and regulating the activity of related proteinases (19–26). In the present study, we isolated a small water-soluble protein from *Pinellia ternata*, investigated its trypsin inhibitory activity and considered the association of antitrypsin activity with antitumour activity. The present study aimed to isolate and purify short-chain peptides with serine proteinase inhibitory activity from the small water-soluble protein components of raw RP, and to study their physicochemical properties and their ability to inhibit the proliferation of poorly differentiated BGC-823 human gastric adenocarcinoma cells *in vivo* and *in vitro*.

## Materials and methods

### Animals

Specific pathogen-free BALB/c nude mice were provided by the Experimental Animal Centre at Shandong University of Traditional Chinese Medicine (Jinan, China). This study was carried out in strict accordance with the recommendations in the Guide for the Care and Use of Laboratory Animals of the National Institutes of Health (2010, Eighth edition). The animal use protocol was reviewed and approved by the Institutional Animal Care and Use Committee of Shandong University (Jinan, China).

### Extraction and separation of RPTI

Fresh *Pinellia ternata* tuber (100 g; ChengWu pinellia planting base, Heze, China) was washed, homogenised in 10-fold its volume of buffer solution for extraction (0.05 mol/l, pH 8.0, Tris HCl) and centrifuged (4,650 × g, 10 min). After rinsing off the floating solid fatty materials, the supernatant was collected and then lyophilised using a lyophiliser (FD-1D-50; Shanghai Bilon Instrument Co., Ltd., Shanghai, China) to obtain the lyophilised crude RP protein powder. The powder was weighed and its TI activity was detected.

The lyophilised powder was redissolved in an appropriate amount of buffer solution for extraction using the stepwise salting-out method. (NH_4_)_2_SO_4_ with 40, 60 and 80% degrees of saturation was gradually added to the solution, which was left to stand at 4°C for 2 h and then centrifuged (8,000 rpm, 10 min). The precipitate from all steps was collected and dialysed with distilled water at 4°C for 36 h; during the dialysis, the dialysate was replaced nine times and the molecular weight of the proteins retained by the dialysis bag was ≥6,000 Da (Pharmacia Biotech). After centrifuging (8,000 rpm, 10 min), the supernatant was lyophilised and weighed. The TI activity was detected and the dialysed and lyophilised (NH_4_)_2_SO_4_ precipitate with the highest activity was designated as the crude RPTI product.

### Protein content determination

The protein content was determined using the Lowry protein assay method with bovine serum albumin (Sigma-Aldrich, St. Louis, MO, USA) as the standard (27).

### Preparation of the affinity carrier

CNBr-activated Sepharose CL-4B (Pharmacia Biotech, Stockholm, Sweden) was coupled with an appropriate amount of trypsin to prepare the affinity carrier based on the manufacturer’s instructions (2010).

### RPTI purification

The crude RPTI product (20 mg) was dissolved in 200 ml balanced buffer solution (0.05 mol/l, pH 8.0, Tris HCl) and centrifuged (4°C, 10,464 × g, 10 min). Following maintenance of the supernatant in the affinity column at 37°C for 1 h, the column was washed with balanced buffer solution containing 1 mol/l NaCl, distilled water and hydrochloric acid solution at pH 2.4. The eluent of the acid solution was collected and was immediately neutralised dropwise with 2.0 mol/l Tris. Following repeated sample loading, the eluent was combined and directly filtered with a Sephadex G-50 chromatographic column (Pharmacia Biotech) and with 0.05 mol/l Tris HCl buffer solution (pH 8.0) as the eluent. The trypsin inhibition activity peak was determined and the protein purity of the activity peak in each tube was detected with SDS-PAGE. The SDS-PAGE index was based on the literature (28). The gel concentration was 12% and Coomassie Brilliant Blue R-250 was used for staining.

### RPTI sequencing

The N-terminal amino acid sequence of RPTI was performed by Kang Biotechnology Compnay of Shanghai (Shanghai, China) using the Edman degradation method with an ABI 491A amino acid sequencer (Applied Biosystems, Foster City, CA, USA) (29).

### Determination of TI activity

Based on the literature (30), trypsin activity (Sigma-Aldrich) and TI activity were detected using BAPNA (Sigma-Aldrich) as the substrate. The sample to be tested was dissolved in 0.80 ml Tris HCl buffer solution (0.05 mol/l, pH 8.0), 0.20 ml bovine trypsin (0.10 mg/ml) was added and the system was maintained at 37°C for 5 min. Subsequently, 2.50 ml BAPNA solution (1 mmol/l) was added, the system was maintained at 37°C for 5 min and then 0.5 ml 33% acetic acid was immediately added to terminate the reaction. The absorbance (A*)* was detected at 410 nm, with the test sample without inhibitor as the control sample. One unit of enzyme activity was defined as the amount of enzyme required to increase A410 by 0.01, whereas one unit of RPTI-inhibitory activity was defined as the amount of enzyme required to decrease A410 by 0.01.

### Cell culture

Dulbecco’s modified Eagle’s medium (DMEM) (Gibco-BRL, Carlsbad, CA, USA) was formulated based on the product description, adjusted to pH 7.2–7.4 with 0.1 mol/l hydrochloric acid and maintained at 4°C. The foetal bovine serum was deactivated at 56°C for 30 min and then maintained at −20°C. Trypsin was diluted to a 2.5 g/l solution with 0.01 mol/l phosphate-buffered saline (PBS) at pH 7.4 and then maintained at 4°C. The poorly differentiated BGC-823 human gastric adenocarcinoma cells (Chinese medicine biotechnology laboratory of Shandong Traditional Chinese Medicine University) were incubated in DMEM containing 10% refined calf serum (Gibco-BRL), 100 U/ml penicillin and 100 μg/ml streptomycin under a 5% CO_2_ atmosphere at 37°C and were subcultured following trypsinisation when the BGC-823 cells adhered to the walls of the flask.

### MTT method

Based on the literature (31), 5 g/l MTT (Sigma-Aldrich) solution was formulated with normal saline, sterilised through filtration with a 0.22-μm filter (Millipore, Billerica, MA, USA), subpacked and then maintained at 4°C. The BGC-823 cells in the logarithmic phase were inoculated into 96-well plates at a density of 1×10^5^ cells/ml and with 100 μl/well and treated following culture for 24 h. Five RPTI concentrations were established: 32, 16, 8, 4 and 2 μg/ml and six dual wells were established for each dose. At 48 and 72 h after treatment, three dual wells were selected and the culture supernatant was discarded through aspiration from the wells. Each well was washed with PBS once and then the supernatant was removed through aspiration. Subsequently, 100 μl complete DMEM and 10 μl MTT solution were added into each well and the plates were incubated for 4 h at 37°C under a saturated 5% CO_2_ atmosphere. The culture was then terminated and the cultural supernatant was carefully discarded by aspiration from the wells. Dimethylsulphoxide (150 μl) was added into each well and agitated for 10 min for full dissolution. The absorbance at 490 nm was determined with an enzyme-labelled instrument (3550 microplate reader; Bio-Rad, Hercules, CA, USA) to compute the cell growth inhibition rate according to the following formula: cell growth inhibition rate (%) = (1 − average A of the treatment group)/average A of the control group × 100]. The IC_50_ of RPTI was calculated using SPSS software, version 13.0 (SPSS, Inc., Chicago, IL, USA).

### Determination of in vivo antitumour activity

BGC-823 cells in the logarithmic phase were washed twice with DMEM and then resuspended in DMEM at a density of 1×10^6^ cells/ml. Subsequently, 0.1 ml of the cell suspension, i.e., 1xl0^5^ BGC-823 cells, was subcutaneously (SC) injected into the back of each BALB/C-nu mouse. Five mice were inoculated, which were regularly observed and fed. After 20 days, the mice were sacrificed, the tumours were dissected and their fibrous capsules were removed. The well-grown tumour tissues were selected, cut into 1-mm^3^ sections (weighing ~40 mg) and added to 0.2 ml normal saline. One section of the tumour tissue was transplanted into the left axilla of each nude mouse. The following day, the inoculated nude mice were randomly assigned into three groups, with 10 mice in each group: The control group (SC injection of normal saline at 10 ml/kg once daily); the cyclophosphamide (CTX) group (SC injection of CTX around the tumour at 20 mg/kg once every two days); and the RPTI groups (SC injection of RPTI at 250, 50 and 10 mg/kg once daily). The treatments were administered for 12 consecutive days. One day after completing the treatments, the mice were weighed and then sacrificed through cervical dislocation. The subcutaneous tumours were dissected and the tumour weights among the groups were compared. The tumour inhibition rate was then calculated. The spleen was collected under sterile conditions and weighed to determine the spleen coefficient: Spleen coefficient = spleen weight (mg)/body weight (g).

### Statistical analysis

The different RPTI treatment groups were compared using SPSS software, a homogeneity test of variance and a t-test. The data were expressed as the mean ± standard deviation. P<0.05 was considered to indicate a statistically significant result.

## Results

### RPTI extraction and separation

Fresh *Pinellia ternata* tuber was ground, homogenised, aqueously extracted and lyophilised to obtain a water-soluble lyophilised protein powder. (NH_4_)_2_SO_4_ precipitates of the total protein of *Pinellia ternata* at all levels were obtained by further using the stepwise salting-out method. The total recovery rate of the precipitate was 96.49%. Analysis indicated that TI activity was mainly concentrated in the 40% (NH_4_)_2_SO_4_ precipitate and the specific trypsin inhibitory activity was 1.83-fold that of the total protein; the recovery of inhibitory activity was 62.38% and the total protein content of the precipitate was 123.67 mg, accounting for 34.13% of the total protein. The majority of the protein precipitated with 60% (NH_4_)_2_SO_4_, accounting for 56.62% of the total protein, and it had partial TI activity; its specific activity was only 0.50-fold that of the total protein. The 80% (NH_4_)_2_SO_4_ solution precipitated the least amount of protein, which exhibited no TI activity (Table I).

### RPTI purification

**Figure 1:**
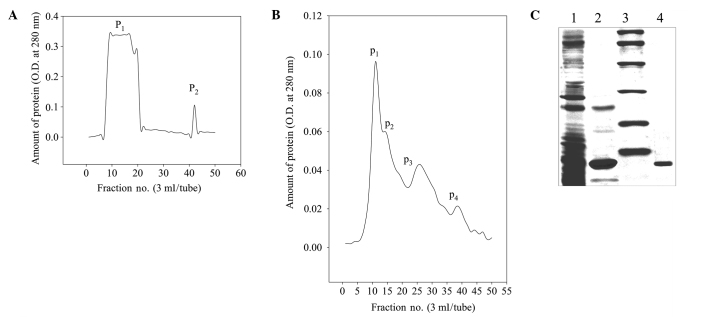
Separation, purification and electrophoresis of RPTI. (A) Sepharose 4B trypsin-affinity chromatography. (B) Sephadex G-50 gel filtration chromatography. (C) SDS-PAGE of RPTI: Lane 1, 40% (NH_4_)_2_SO_4_ precipitation; 2, affinity chromatography; 3, molecular weight markers (from top to bottom: 97.4, 66.2, 43.0, 31.0, 20.1 and 14.4 kDa); and 4, gel filtration. OD, optical density; RPTI, Rhizoma Pinelliae trypsin inhibitor.

The 40% (NH_4_)_2_SO_4_ precipitate was designated as the crude RPTI product and subjected to trypsin-Sepharose 4B affinity chromatography to form two protein peaks (A_280 nm_; Fig. 1A). P_1_ was a saliferous elution peak that exhibited no TI activity and P_2_ was an affinity adsorption activity peak, which was further separated using Sephadex G-50 to form four protein peaks (Fig. 1B). The P_1_ and P_2_ formed by Sephadex G-50 separation were TI activity peaks, which were mainly concentrated in tubes 10–16. The 12% SDS-PAGE purity detection indicated that only the components in tubes 10–12 (the first half of P_1_) exhibited a single protein band and were estimated to have >90% purity. The eluents of the samples in tubes 10–12 following repeated sample loading were collected and combined to obtain purified RPTI. Fig. 1C shows that the crude RPTI protein had numerous bands and a complex composition. Further affinity chromatography removed the majority of the hybrid proteins and only left two clear main bands. More uniform main bands of RPTI were obtained following the gel filtration, with an apparent relative molecular weight of ~14 kDa. The activity recovery during each purification step is listed in Table II. Following the affinity chromatography, the specific inhibitory activity increased by 3.45-fold. The Sephadex G-50 separation produced a homogenous component. The activity recovery was 24.40% and 6.02-fold purified RPTI was obtained.

### N-terminal amino acid sequence and homology

Following SDS-PAGE and the transmembrane filtration, the N-terminal amino acid sequence of the pure RPTI was determined. The first six amino acid residues at the N-terminal of the RPTI in the present study were 1-DPVVDG-6. The BLAST database of the NCBI indicated that the first six amino acids in the N-terminal sequence of RPTI are the same as those of arrowhead proteinase inhibitors A and B, with serine replacing glycine at the sixth position being the only difference. The RPTI sequence was highly homologous to those of the arrowhead proteinase inhibitors (>80%; Table III).

### Inhibitory effect of RPTI on trypsin activity

A 10-μg/ml RPTI solution was formulated with 0.05 mol/l Tris HCl (pH 8.0) buffer solution and successively diluted to 5.00, 2.50, 1.25 and 0.625 μg/ml. The inhibitory activity of RPTI on trypsin was determined using the following method: 0.80 ml diluted sample solution under test is added to 0.20 ml bovine trypsin and incubated at 37°C for 5 min. Then 2.50 ml BAPNA solution was added and reacted at 37°C for 5 min, the reaction was immediately terminated and added to 0.5 ml 33% acetic acid solution, the absorbance (A) was detected at 410 nm, with the test sample without inhibitor as the control sample. Table IV shows that at 0–2.5 μg/ml RPTI, the trypsin inhibition activity had a good linear association with the concentration of RPTI. The linear regression equation was Y = −25.709X + 94.862 (R^2^ = 0.9626). The RPTI concentration required to completely inhibit 20 μg of trypsin was 3.69 μg/ml and the inhibition ratio (mass) of RPTI to trypsin was 1:6.78, with a molar inhibition ratio of 1:1.69.

### Inhibitory effect of RPTI on BGC-823 cell proliferation in vitro

Table V shows that RPTI significantly inhibited the proliferation of BGC-823 cells and that the inhibition was concentration-dependent. The IC_50_ of RPTI calculated using logistic regression was 16.96 μg/ml within 48 h after treatment and 9.61 μg/ml within 72 h after treatment.

**Figure 2:**
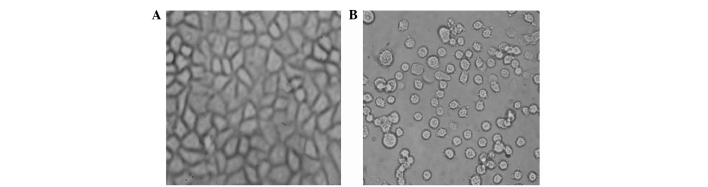
Influence of RPTI on BGC-823 cell shape (magnification, ×100). (A) Control; (B) RPTI treatment (10 μg/ml RPTI, 72 h after addition to the cells). RPTI, Rhizoma Pinelliae trypsin inhibitor.

The BGC-823 cells were treated with 10 μg/ml RPTI (Fig. 2). After 72 h, the changes in cell shape were observed under an inverted microscope. The BGC-823 cells in the RPTI group were less shrunken and were significantly reduced in number compared with those of the control group, with numerous suspended cells being dead. The cells exhibited nuclear pyknosis and rough cytoplasms and underwent less fission. The control cells had regular shapes and good adherent growth.

### Activity of RPTI against BGC-823 cells in vivo

Table VI shows that subcutaneous RPTI administration around the tumour significantly inhibited the growth of the transplanted BGC-823 cells and the inhibition was dose-dependent. The tumour inhibition rates of the high-dose and medium-dose groups were significantly higher than that in the control group, but was lower than that of the CTX positive control group. The tumour inhibition effect in the low-dose group was not significantly different from that in the control group. RPTI was not observed to have a significant effect on the spleen coefficient of the mice.

## Discussion

*Pinellia ternata* tuber is used for the clinical treatment of various neoplastic diseases alone or combined with other Chinese medicines (5–8). Studies have suggested that the *Pinellia* protein significantly inhibits the proliferation of ovarian cancer cells (11). TIs are widely present in plants and are widely accepted in the medical field as potential cancer preventive agents. In the present study, a small protein from *Pinellia ternata* tuber was isolated through separation methods, demonstrated that this component has certain trypsin inhibitory activity and inferred that RPTI may be a component of *Pinellia ternata* that has antitumour activity. To the best of our knowledge, the present study is the first to isolate and purify RPTI. The purified RPTI showed a single band under SDS-PAGE, with a molecular weight of 14 kDa. Its N-terminal amino acid sequence was DPVVDG, which is highly homologous to that of arrowhead serine proteinase inhibitors. The inhibition rate (mass) of RPTI to the trypsin activity was 1:6.78. RPTI markedly inhibited trypsin activity and its inhibition constant Ki was significantly lower than the Km of trypsin (data not shown). Therefore, RPTI was considered as a strong serine proteinase inhibitor.

The present study preliminarily demonstrates that RPTI has a significant inhibitory effect on BGC-823 cell proliferation in vitro and on the tumour growth of transplanted BGC-823 cells. The tumour inhibition was concentration- and dose-dependent. Consequently, RPTI has significant antitumour activity. A key step in tumour cell growth and infection is extracellular matrix (ECM) degradation. RPTI likely exerts its antitumour effects by combining with the serine proteinase or a specific receptor on the external surface of BGC-823 cell membranes through different approaches. Thus, the proteinase loses its ability to hydrolyse the ECM, preventing BGC-823 cell invasion and tumour growth.

During the separation and purification of RPTI using affinity chromatography, the samples were maintained at 37°C for 1 h after loading. The column was then washed with a highly concentrated salt solution (1 mol/l NaCl) to wash off the majority of nonspecific binding hybrid proteins. The bonding RPTI ingredients were eluted with aqueous acid so that the purity of the RPTI, separated through affinity chromatography with trypsin as the ligand, was significantly improved. The P_1_ and P_2_ activity peaks generated through Sephadex G-50 filtration were not completely separated and the ingredients in tubes 10–16 were detected to have clear TI activity. Only the first half of the P_1_ peak, i.e., the proteins in tubes 10–12, showed a uniform band in the SDS-PAGE. Following further electrophoresis and transmembrane separation, the main band of the purified RPTI ingredient was cut off for sequencing to ensure the purity of the RPTI.

The serine proteinase inhibitor separated from the tuber of *Pinellia ternata* has promising potential for application in antitumour therapy. The aim of further studies is to design degenerate primers according to the RPTI N-terminal amino acid sequence, clone its cDNA sequence, elucidate the complete gene expression of RPTI and determine its mechanism of action.

## Figures and Tables

**Table I:** Isolation of RPTI crude protein by salt fractionation with (NH_4_)_2_SO_4_ and determination of its inhibitory activity.

Component	Total protein (mg)	Total inhibitory activity (U)	Inhibition recovery (%)	Specific inhibitory activity (U/mg)
Total protein	362.33	34750	100.00	95.91
40% (NH_4_)_2_SO_4_ precipitate	123.67	21677	62.38	175.81
60% (NH_4_)_2_SO_4_ precipitate	205.14	9883	28.44	48.18
80% (NH_4_)_2_SO_4_ precipitate	20.81	0	0.00	0.00
Total (NH_4_)_2_SO_4_ precipitate	349.62	31560	90.82	90.27

RPTI, Rhizoma Pinelliae trypsin inhibitor.

**Table II:** Purification steps of RPTI.

Purification step	Total protein (mg)	Total inhibition activity (U)	Specific inhibition activity (U/mg^−1^)	Inhibition recovery (%)
40% (NH_4_)_2_SO_4_ precipitate	20.00	3516.20	175.81	100.00
Affinity chromatography	2.19	1326.50	605.71	37.73
Sephadex G-50 gel filtration	0.81	857.80	1059.01	24.40

RPTI, Rhizoma Pinelliae trypsin inhibitor.

**Table III:** N-terminal amino acid sequence of five protease inhibitors and their homology comparison results.

ID	Name	N-terminal amino acid sequence	Homology (%)
This study	RPTI	1 DPVVDG 6	5/6 (83.33)
BAA02972.1	Precursor of arrowhead proteinase inhibitor A	25 DPVVDS 30	5/6 (83.33)
BAA02973.1	Precursor of arrowhead proteinase inhibitor B	25 DPVVDS 30	5/6 (83.33)
1818181A	Arrowhead proteinase inhibitor A	1 DPVVDS 6	5/6 (83.33)
1208229A	Arrowhead proteinase inhibitor B	1 DPVVDS 6	5/6 (83.33)

RPTI, Rhizoma Pinelliae trypsin inhibitor.

**Table IV:** Inhibitory activity of RPTI on trypsin.

RPTI concentration (μg/ml)	A410 nm (mean ± SD, n=3)	Residual enzyme activity (%)	Enzyme activity inhibition rate (%)
0.000	0.218±0.014	100.00	0.00
0.625	0.166±0.017^a^	76.15	23.85
1.250	0.123±0.013^a^	56.42	43.58
2.500	0.075±0.01^a^	34.40	65.60
5.000	0.047±0.009^a^	21.56	78.44

a P<0.01, compared with the control group.

RPTI, Rhizoma Pinelliae trypsin inhibitor.

**Table V:** Inhibitory effect of RPTI on the *in vitro* proliferation of BGC-823 cells.

	48 h after addition of RPTI		72 h after addition of RPTI	
		
RPTI (μg/ml)	Absorbance (A)	Inhibition rate (%)	Absorbance (A)	Inhibition rate (%)
0	0.163±0.014	-	0.178±0.017	-
2	0.147±0.001	9.82	0.152±0.013^a^	14.42
4	0.131±0.007^a^	19.63	0.132±0.011^a^	25.78
8	0.109±0.008^a^	33.13	0.099±0.009^a^	44.26
16	0.082±0.006^a^	49.69	0.060±0.007^a^	66.53
32	0.059±0.005^a^	63.80	0.037±0.008^a^	79.04

Absorbance values are the mean ± SD, n=10.

a P<0.01, compared with the control (0 μg/ml) group.

RPTI, Rhizoma Pinelliae trypsin inhibitor.

**Table VI:** Tumour inhibition rate of RPTI on BGC-823 cells in tumour-bearing mice and the spleen coefficient.

Group	Dose (mg/kg)	Tumour weight (g)	Tumour inhibition rate (%)	Spleen coefficient (mg/g)
Control	-	1.44±0.26	0.00	5.64±0.83
CTX	20	0.28±0.08^a^	80.56	5.25±0.75
High dose of RPTI	250	0.41±0.11^a^	71.53	5.83±1.06
Medium dose of RPTI	50	0.96±0.19^a^	33.33	5.78±0.96
Low dose of RPTI	10	1.18±0.23	18.06	5.67±0.87

Conducted in triplicate. Tumour weight and spleen coefficient values are the mean ± SD.

a P<0.01, compared with the control group.

RPTI, Rhizoma Pinelliae trypsin inhibitor; CTX, cyclophosphamide.
